# Zinc Oxide Administration Relieves the Diarrhea of ETEC K88-Infected Piglets by Reducing Ileal Apoptosis and Maintaining Gut Microbial Balance

**DOI:** 10.3390/vetsci12020115

**Published:** 2025-02-02

**Authors:** Yanyan Zhang, Jiale Liu, Muzi Li, Yi Dong, Zongyun Li, Dan Yi, Tao Wu, Lei Wang, Di Zhao, Yongqing Hou

**Affiliations:** Engineering Research Center of Feed Protein Resources on Agricultural By-Products, Ministry of Education, Hubei Key Laboratory of Animal Nutrition and Feed Science, Wuhan Polytechnic University, Wuhan 430024, China

**Keywords:** gram-negative enterotoxin piglets, ileal apoptosis, pathogen-induced diarrhea, ETEC K88, diarrhea, gut health, gut microbial

## Abstract

The impact of ZnO as a feed additive on growth-performance and intestinal function of *Enterotoxigenic Escherichia coli* (ETEC) K88-infected piglets remains unclear. Fecal scores of piglets in ETEC group were significantly increased compared to control group. ETEC K88 significantly damages the small intestine, including a reduction in villus height in the jejunum, duodenum, and ileum, and a decrease in total superoxide dismutase activity in the jejunum and catalase activity in the ileum and jejunum. Compared to control group, ETEC K88 infection significantly elevated the mRNA level of gene *IL-1β* and the level of ileal epithelial cell apoptosis. ZnO administration significantly alleviated these negative effects and improved the antioxidative capability of the ileum. Moreover, ZnO supplementation alleviated the imbalance of gut microbiota by restoring the reduced amount of *Enterococcus* and *Lactobacillus* in the jejunum, *Clostridium* in the ileum, and *Lactobacillus* in the cecum, as well as the increased amount of total eubacteria in the ileum and *Enterococcus* in the cecum induced by the ETEC K88 infection. In conclusion, ZnO administration can reduce the diarrhea of piglets infected with ETEC K88 by reducing the structural damage of the intestine, attenuating intestinal oxidative stress and epithelial cell apoptosis, and modulating the gut microbiota.

## Simple Summary

Zinc plays a crucial effect on intestinal health in animals. The present study explored the impact of zinc oxide (ZnO) on the blood biochemical indicators, intestinal morphology, antioxidant indicators, inflammation genes, and gut microbiota of ETEC K88-infected piglets. ZnO (100 mg/kg BW) administration can reduce the diarrhea of ETEC K88-infected piglets by reducing intestinal structural damage, attenuating intestinal oxidative stress and epithelial cell apoptosis, and modulating the gut microbe. The study elucidated the important function of ZnO as a feed additive in piglet intestinal health and provided a theoretical basis for the application of ZnO to improve the intestinal function of piglets infected with ETEC K88.

## 1. Introduction

The intestine mainly comprises the mucosal layer and the lamina propria including epithelial cells, immune cells, and tight junctions. It is not only an important part of absorbing and digesting nutrients but also the largest immune organ [1,2]. A healthy intestine is critical for the disease defense, overall metabolism, physiology, and growth of piglets [3] The intestinal epithelial cells serve to absorb nutrients, which play a crucial role in preventing antigens from entering blood circulation by involving in the formation of tight junctions [4]. Therefore, the intact intestine is important for piglets to maintain normal life activities [5]. The small intestine as the longest digestive system is essential for nutrient digestion and absorption. In addition, various bacteria parasitize the intestine, which is divided into beneficial bacteria, intermediate bacteria, and harmful bacteria. When the intestinal environment is healthy, these three types of bacteria are in a dynamic equilibrium state [6]. The gut microbiota is important for piglets to develop the immune system and structure of the intestine [7]. However, as intestinal function and structure are not yet fully developed, piglets are susceptible to the stimulation of various external environmental factors, which will influence intestinal function and structure and lead to intestinal bacterial dysbiosis [8,9,10,11]. An imbalance of gut microbiota causes the translocation of gut microorganisms, resulting in the weakened function of the intestinal barrier, excessive production of pro-inflammatory cytokines, and excessive activation of the intestinal mucosal immune system, which in turn together promotes the development of inflammatory bowel disease [12,13,14]. Therefore, it is important for the growth and development of piglets to maintain the balance of gut microbiota.

However, the pathogenic microorganisms seriously hinder the healthy development of piglets. ETEC is one of the common bacterial pathogens that seriously threaten the intestinal health of piglets [15]. ETEC can produce one or several enterotoxins causing secretory diarrhea [16]. ETEC mainly causes diarrhea in piglets, leading to higher incidence and mortality rates, reduced growth performance, and significant economic losses in the pig industry. A study has shown that ETEC is capable of causing a systemic or localized immune response, which will cause the excessive release of inflammatory cytokines and damage intestinal cells or tissues [17]. Adhesins are proteins that can play a significant role in bacterial colonization and are mainly composed of proteins or phospholipids, usually found in the cell walls and pili on the surface of bacteria. The common pili adhesins in ETEC include F4 (K88), F5 (K99) and others. One of the biggest threats to piglet intestinal health is ETEC K88. Firstly, ETEC adheres to the intestinal epithelial cell receptors of piglets by adhesins. Subsequently, ETEC can avoid shedding caused by intestinal peristalsis and stably colonize. Finally, ETEC destroys the intestinal epithelial cells by secreting heat-labile enterotoxins (LT)and heat-stable enterotoxins (ST), which will cause water and electrolyte disorders in the body, ultimately leading to diarrhea [18,19,20]. Therefore, it is important to find green nutritional modifiers to prevent or treat intestinal damage in piglets caused by ETEC K88.

Zinc (Zn) is a key trace element in animal nutrition, which not only participates in cell proliferation and differentiation but also maintains cell membrane integrity and regulates protein metabolism in the body [21,22]. When Zn is deficient, animals usually show symptoms such as decreased feed intake, reduced feed conversion efficiency, and accompanying [23]. Zn can be added to feed in many ways, with ZnO being the most common, and ZnO is important for weaned piglets to control diarrhea and improve growth performance. Previous study has shown that ZnO in feed improves several digestive enzyme activities in the pancreatic tissue of pigs, improves intestinal morphology, and consequently improves the digestion and absorption of nutrients [24,25]. Cytokines can be directly or indirectly involved in immune-inflammatory response reactions. Previous research reports showed that ZnO treatment improved the expression level of inflammatory factors (TGF-β) by using piglet intestinal epithelial cells (IPEC-J2) infected with *E. coli* F4 as an in vitro research model, suggesting that ZnO treatment inhibited the inflammatory response caused by ETEC K88 infection [26,27]. Recent reports have shown that ZnO plays a very important role in animal production, which not only can improve immunity function and enhance antioxidant properties, but also reduce diarrhea rate and improve growth performance. However, the influences of ZnO on the damage of the intestine of piglets infected with ETEC are not fully understood. The inclusion of ZnO in medicated pig feed, which is utilized to treat neonatal diarrhea and other health issues in piglets, is prohibited in several countries, including those in the European Union, due to concerns regarding environmental and public health, but in China, the maximum allowable dose of zinc oxide for use in piglets within the first two weeks after weaning is 1600 ppm. The purpose of this study is to evaluate the impact of ZnO (100 mg/kg BW) on intestinal function and growth performance in ETEC K88-infected piglets.

## 2. Materials and Methods

### 2.1. Ethics Statement

The animal experiments were approved by the Animal Care and Use Committee of Wuhan Polytechnic University (approval code WPU201910001), and conducted according to the Chinese Guidelines for Animal Welfare and Experimental Protocol in this study.

### 2.2. Experimental Animals and Diet

Twenty-four piglets (Duroc × Landrace × Yorkshire) that were weaned at 7 days of age were selected (Seven-day-old piglets are at a critical stage of growth and development, when the immune system and digestive systems of piglets are developing rapidly, and pigs at this stage are chosen as experimental animals to better observe and study the effects of external factors on their health and growth after weaning). After three days of adaptation, the piglets were raised individually in a stainless steel metabolic cage (1.0 × 1.5 m^2^) that had been thoroughly cleaned and disinfected. The room temperature was maintained between 29–34 °C. Piglets in this study can freely drink and eat from the feeder and nipple-type drinker that was included in each cage. The basal diet was a milk replacer with a digestible energy content of ≥3400 kcal/kg supplied by Shanghai Gaode Feed Co., Ltd. (Shanghai, China). The nutrient composition of the diets is the same as that of Zeng et al. [28]. The piglets were fed the basic diet at 8:30, 12:00, 15:00, 18:00, and 21:00 daily during the experimental period. The dry matter to water ratio of the experimental diet was 1:5 when dissolved in warm water at approximately 45 °C. Fecal scores were assessed five times daily following the ETEC K88 challenge. Four categories for pig feces were established: 0 score (normal), 1 score (soft feces), 2 score (mild diarrhea), and 3 score (severe diarrhea) [29].

### 2.3. Experimental Design

Twenty-four healthy 7-day-olds were purchased from a commercial pig farm. Piglets (1.79 ± 0.24 kg) half-male and half-female piglets were randomly assigned to three treatment groups: (1) the Control group; (2) the ETEC group; (3) the ZnO + ETEC group. During the acclimatization period, piglets in each group were allowed to feed freely. From days 1 to 6 of the experiment, the ZnO + ETEC group was administered ZnO (100 mg/kg BW, ZnO was dissolved in PBS to form 50 mg/mL solution, and the instillation dose was converted to 2 mL/kg BW) daily at 5 p.m. The control and ETEC groups received the same volume of PBS orally (2 mL/kg BW). To determine the effect of ZnO as a feed additive on effects on intestinal function and growth performance in ETEC K88-infected piglets. On Day 5 of the experiment, the piglets in the control group were given 2 mL of PBS orally, and piglets in the ETEC and ZnO + ETEC groups received 2 mL of PBS containing ETEC K88 (2.5 × 10^9^ CFU/mL). The ETEC K88 strain was gifted by the Guangdong Academy of Agricultural Sciences. The organism was grown in Luria-Bertani (LB) medium at a temperature of 37 °C for 9 h. When the optical density at 600 nm (OD_600_) reached 0.9, ETEC K88 was harvested by centrifugation at 6000× *g* for 10 min and then resuspended in PBS to achieve a final concentration of 2.5 × 10^9^ CFU/mL. From Days 1 to 6 of the experiment, the consumption of feed (g) and the body weight (g) were measured to assess the piglet growth performance and to determine the average daily feed intake (g/d). To ensure consistency in dosing, fresh ETEC K88 and ZnO solutions were prepared daily. Piglets received an oral solution of D-xylose solution (10% D-xylose, 1 mL/kg BW) and were weighed on Day 7 of the experiment. Blood samples were obtained to assess the capacity for intestinal absorption and the integrity of mucosal an hour later [30]. After blood collection, sodium pentobarbital anesthetic (50 mg/kg BW) was administered via intravenous injection, and after the piglets became unconscious, the anesthetized piglets were put to death and the small intestine was collected.

### 2.4. Blood Sample Collection

Anterior vena cava blood samples were collected 1 h after D-xylose administration via a vacuum blood collection system in Sodium Heparin Anticoagulation Vacuum Blood Collection Tubes and EDTA Anticoagulation Vacuum Blood Collection Tubes (Becton-Dickinson Vacutainer System, Franklin Lake, NJ, USA), separate collection of plasma and whole blood. The plasma was then extracted by centrifuging for 15 min at 1200× *g* at 4 °C within half an hour of blood collection [31,32]. Relative to the plasma volume and metabolic capacity of each piglet, the infusion of D-xylose is low and does not affect biochemical parameters. The plasma samples were kept in an ultra-low temperature freezer (−80 °C) until it was analyzed.

### 2.5. Intestinal Samples

The abdomens of the piglets were split apart right away, exposing the whole digestive system from the sternum to the pubis [31,33]. After being removed from the mesentery, the small intestine was put on a cold stainless-steel dish. Approximately 3 cm intestinal segments were removed from the mid-section of the duodenum, jejunum, and ileum and preserved in 4% paraformaldehyde for morphometric measurements. After being handled on ice, ten-centimeter samples of the mid-ileum were cut lengthwise, carefully cleaned with cold saline, dried using filter paper, cut into smaller pieces, and then quickly frozen in liquid nitrogen before being stored at −80 °C for analysis [34]. After sacrifice, all samples were gathered in less than 20 min.

### 2.6. Intestinal Morphological

The tested intestinal segment was fixed for 24 h in 4% paraformaldehyde, and then hematoxylin and eosin staining were used to create a paraffin section with a thickness of 4 µm [35]. Each pig was assigned one slice, and eight villus per slice were selected for measurement and subsequently averaged. An optical microscope (Olympus BX-41 TF, Tokyo, Japan) and cellSens Standard software were used to measure the crypt depth, villus height, and villus width of the duodenum, ileum, and jejunum in accordance with the protocol Uni et al. described [36].

### 2.7. Determination of the Biochemical Indicators in Plasma, Activities of Diamine Oxidase, and the Content of D-Xylose

Plasma biochemical indices including Aspartate aminotransferase (AST), Alkaline Phosphatase (ALP), High-Density Lipoprotein (HDL), Creatinine (CREA), Blood Urea Nitrogen (BUN), Gamma-Glutamyl Transferase (GGT), and Creatine Kinase (CK) were calculated using the Hitachi 7060 (Hitachi, Tokyo, Japan) automated biochemical analyzer. The activity of diamine oxidase (DAO) in plasma and the concentration of D-xylose were measured by utilizing specific kits (Jiancheng Bioengineering Institute, Nanjing, China).

### 2.8. Blood Cell Counts

The whole blood collected during the experiment was subjected to blood cell counting using the ADVIA 2120i blood analysis system (Siemens Healthcare Diagnostics, USA), including White Blood Cell (WBC), Cell Hemoglobin Concentration Mean (CHCM), Mean Corpuscular Hemoglobin Concentration (MCHC), Hemoglobin Distribution Width (HDW), Lymphocytes (LYMPH), Monocytes (MONO), Basophil (BASO), Neutrophils (NEUT), Large Unstained Cells (LUC).

### 2.9. Intestinal and Plasma Antioxidant Indices

The plasma, ileum, jejunum, and duodenum underwent pre-treatment following previous protocols [37]. The activities of glutathione peroxidase (GSH-Px), superoxide dismutase (T-SOD), and catalase (CAT), along with the levels of malondialdehyde (MDA) and hydrogen peroxide (H_2_O_2_) in intestinal samples were assessed using commercial reagent kits (Nanjing Jiancheng Biotechnology Research Institute, Nanjing, China).

### 2.10. Assessment of Apoptosis in the Ileum Epithelium

The TUNEL cell apoptosis detection kit (FITC, MK1027) acquired by BOSTER Biotechnology Company was utilized in this investigation to evaluate the effect of ZnO supplementation on the apoptosis of intestinal epithelial cells in piglets infected with ETEC K88. The ileum samples were prepared using the previous protocol [37].

### 2.11. Determination of Gut Microflora

The bacterial DNA from the contents of the jejunum, ileum, and cecum was extracted and purified by using the QIAamp DNA Stool Mini Kit (Qiagen, Germantown, MD, USA) according to the operation instructions. The digital PCR was utilized to identify gut microbiota composition in the contents of cecum (n = 8) [38], ileum (n = 8), and the jejunum (n = 8) [38]. Table 1 shows the primers used in this study.

### 2.12. Quantitative PCR

TRIzol reagent (Takara, Dalian, China) was used to extract total RNA of about 100 mg of jejunum or ileum samples, and a NanoDrop^®^2000 spectrophotometer (Thermo Scientific, Waltham, MA, USA) was used to measure RNA concentration. The nucleic acids were regarded as pure if the OD_260_/OD_280_ ratio fell within the range of 1.8 to 2.2, indicating a purity greater than 90% [39]. To verify that the RNA is intact, 1% agarose gel electrophoresis was used [40]. The PrimeScript ^®^ RT reagent Kit with gDNA Eraser (Takara, Dalian, China) was used to carry out the reverse transcription synthesis of cDNA. Lastly, a 7500 Fast Real-Time PCR System (Applied Biosystems, Foster City, CA, USA) was used to quantify the mRNA levels of the intestinal genes. The internal reference gene was RPL4, and Table 2 displayed the primer sequences. These results were analyzed by using the 2^−∆∆Ct^ method outlined [34].

### 2.13. Statistical Analysis

This study utilized one-way analysis of variance and the Duncan multiple-range test to assess all experimental data. All data was expressed as mean ± SD SPSS software (Version 17.0, SPSS Inc., Chicago, IL, USA). The weighted averages of the fecal scores were analyzed by using the χ2 test. *p* < 0.05 was deemed statistically significant. GraphPad Prism 9 was used to generate the bar graphs.

## 3. Results

### 3.1. ZnO Administration Attenuated the Increase of Fecal Scores and the Plasma Activity of Diamine Oxidase That Was Triggered by the ETEC-K88 Challenge in Piglets

Compared to the control group, ZnO administration was ineffective on ADFI in the ZnO + ETEC group (Supplementary Figure S1A) during the first four days of the trial. Compared to the control group, ADFI in the ETEC group was significantly decreased (Supplementary Figure S1A) during Days 5 and 6 of the study, and ZnO administration had no significant effect on the ETEC infection-caused decrease of ADFI in the ZnO + ETEC group (Supplementary Figure S1A). Among the three groups, no significant difference was observed in the body weight (BW) of piglets (Supplementary Figure S1B) on Day 1 of the trial. However, compared to the control group, ETEC K88 infection tended (*p* = 0.164) to decrease the BW of piglets in the ETEC group, there was no dramatic difference in BW between the ETEC group and ZnO + ETEC group on Day 7 of the trial (Supplementary Figure S1B).

**Figure 1:**
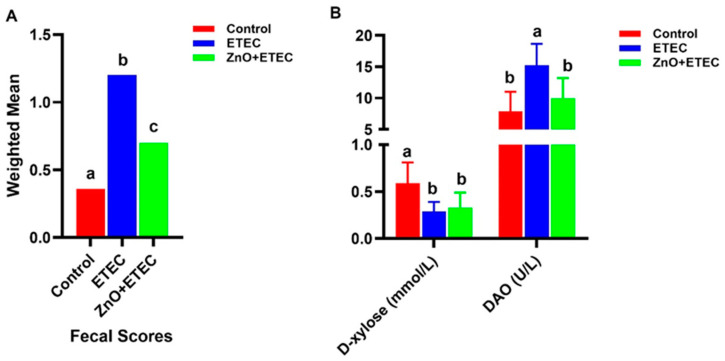
The effects of ZnO administration on the fecal scores and DAO activity of piglets infected with ETEC K88. (**A**) Effect of ZnO administration on fecal scores of ETEC K88-infected piglets. (**B**) Effect of ZnO administration on D-xylose content and DAO activity in ETEC K88-infected piglets. Data are shown as mean ± SD (n = 8), and mean values following different letters at the top of the a, b, and c plots signify statistically significant differences (*p* < 0.05).

Compared to the control group, fecal scores were dramatically higher in the ETEC group on Days 5–6 of the trial, while ZnO administration dramatically alleviated the piglet fecal scores in the ZnO + ETEC group (Figure 1A). In addition, the content of D-xylose in plasma was significantly decreased after ETEC K88 infection. The activity of the plasma DAO was also dramatically increased after ETEC K88 infection, while, compared with that in the ETEC group, ZnO administration dramatically decreased the activity of the plasma DAO in the ZnO + ETEC group (Figure 1B).

### 3.2. The Effects of ZnO Administration on the Blood Biochemical and Hematological Indices in the Piglets Infected with ETEC K88

**Figure 2:**
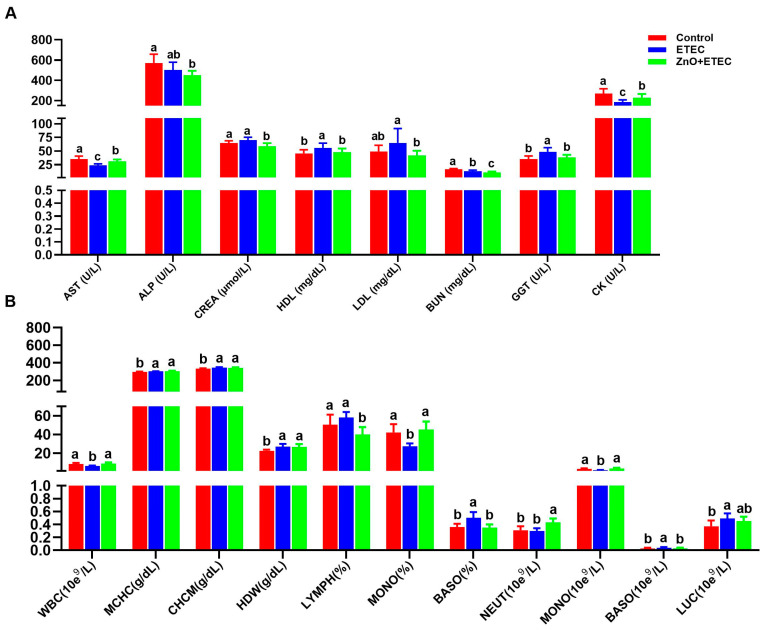
The effects of ZnO administration on hematological parameters and blood biochemical in ETEC K88-infected piglets. (**A**) Effect of ZnO administration on the plasma biochemical parameters in the ETEC K88-infected piglets. (**B**) Effect of ZnO administration on the blood hematological parameters of the ETEC K88-infected piglets. Data are shown as mean ± SD (n = 8), and mean values following different letters at the top of the a, b, and c plots signify statistically significant differences (*p* < 0.05).

Compared to the control group, a significant reduction in BUN levels as well as activities of AST and CK in piglets were detected in the ETEC group, whereas levels of HDL and GGT activity showed a significant increase after ETEC K88 infection (Figure 2A). ZnO administration significantly enhanced the activities of CK and AST, while dramatically decreasing the levels of CREA, HDL, LDL, BUN, and the activity of GGT in piglets in the ZnO + ETEC group compared with the ETEC group (Figure 2A). Compared to the control group, the percentage of MONO and the amount of WBCs, whereas the amounts of BASO and LUC, along with the levels of HDW, MCHC, and CHCM, showed a significant increase in piglets belonging to the ETEC group after ETEC K88 infection (Figure 2B). Conversely, ZnO administration dramatically alleviated the decrease in the counts of MONO and WBC, as well as the elevation in the number of BASO in the ZnO + ETEC group (Figure 2B).

### 3.3. ZnO Administration Significantly Reduced the ETEC K88 Infection-Caused Intestinal Structural Damage in Piglets

**Figure 3:**
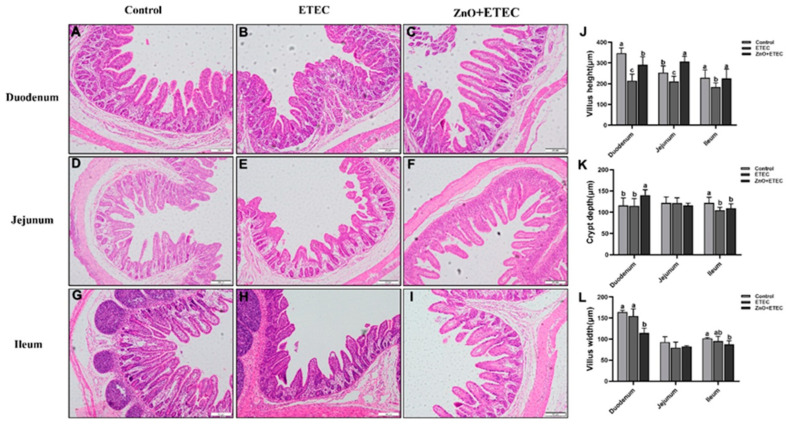
The effects of ZnO administration on intestinal morphometry of ETEC K88-infected piglets. (**A**–**I**) shows H&E staining of ileum, jejunum, and duodenum tissues. H&E: Hematoxylin-eosin staining; Scale bar: 200 μm. (**J**) Villus height. (**K**) Crypt depth. (**L**) Villus width. Data are shown as mean ± SD (n = 8), and mean values following different letters at the top of the a, b, and c plots signify statistically significant differences (*p* < 0.05).

Further results showed that infection with ETEC K88 caused significant harm to the intestinal villi of piglets, which resulted in atrophy and even shedding of intestinal villi in piglets (Figure 3B,E,H) compared with the control group (Figure 3A,D,G). ZnO administration significantly alleviated the ETEC K88 infection-caused damage to the villi of intestine (Figure 3C,F,I). In addition, compared to the control group, measurement results showed that villus height in the jejunum, ileum, and duodenum was dramatically reduced in the ETEC group (Figure 3J), and the crypt depth in the ileum was dramatically reduced after ETEC K88 infection (Figure 3K), while ETEC K88 had no significant impact on villus width in the ileum, jejunum, and duodenum (Figure 3L). However, the villus heights in the ileum, jejunum, and duodenum of ETEC K88-infected piglets were significantly enhanced after ZnO administration (Figure 3J), but also significantly increased the crypt depth in the duodenum (Figure 3K), whereas the villus width of the duodenum was significantly decreased (Figure 3L).

### 3.4. ZnO Administration Increased the Antioxidant Activity in Piglets Infected with ETEC K88

**Figure 4:**
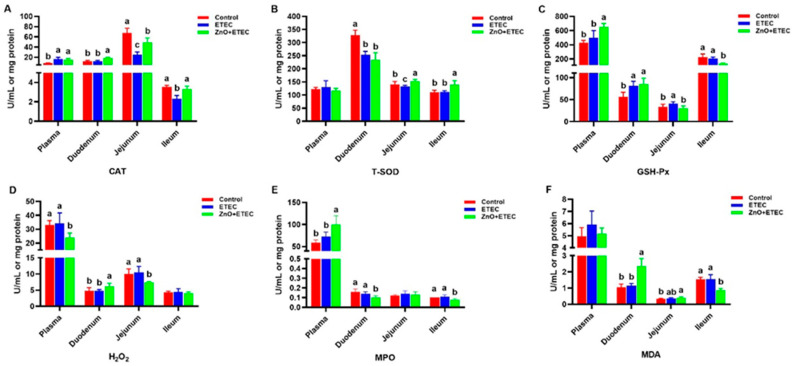
The impact of administration of ZnO on the intestinal antioxidant capacity of piglets infected with ETEC K88. (**A**) CAT activity. (**B**) T-SOD activity. (**C**) GSH-Px activity. (**D**) H_2_O_2_ content. (**E**) MPO activity. (**F**) MDA content. CAT, Catalase; T-SOD, Total Superoxide Dismutase; GSH-Px, Glutathione peroxidase; H_2_O_2_, Hydrogen peroxide; MPO, Myeloperoxidase; MDA, Malondialdehyde. Data are shown as mean ± SD (n = 8), and mean values following different letters at the top of the a, b, and c plots signify statistically significant differences (*p* < 0.05).

Compared with the control group, the activities of CAT in the ileum and jejunum, and the activity of T-SOD in the jejunum and duodenum were significantly decreased, but the activities of GSH-Px in the duodenum and jejunum and plasma CAT were significantly increased after ETEC K88 infection (Figure 4A–C). However the ZnO + ETEC group had higher (*p* < 0.05) CAT activity in the ileum, jejunum, and duodenum, higher (*p* < 0.05) T-SOD activity in the ileum and jejunum, higher (*p* < 0.05) plasma and duodenum GSH-Px activities, higher (*p* < 0.05) plasma MPO activity, the concentrations of H_2_O_2_ and MDA in the duodenum, whereas there was lower (*p* < 0.05) H_2_O_2_ concentration in the jejunum and plasma, lower (*p* < 0.05) MPO activity in the ileum and duodenum, lower (*p* < 0.05) MDA levels in the ileum, and a trend towards lower H_2_O_2_ concentrations in the ileum (*p* > 0.05) (Figure 4A–F) compared with the ETEC group.

### 3.5. ZnO Administration Reduced the ETEC K88-Caused Ileal Inflammatory Response in Piglets

**Figure 5:**
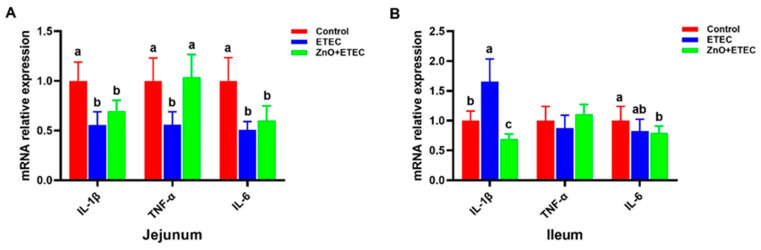
The effect of ZnO administration on the mRNA expression levels of the associated genes in the jejunum and ileum of piglets infected with ETEC K88. (**A**) jejunum; (**B**) ileum. Data are shown as mean ± SD (n = 8), and mean values following different letters at the top of the a, b, and c plots signify statistically significant differences (*p* < 0.05).

Compared with the control group, the mRNA levels of *IL-6*, *TNF-α*, and *IL-1β* in the jejunum were dramatically downregulated in the ETEC group (Figure 5A). The mRNA level of *IL-1β* was dramatically upregulated in the ileum after ETEC K88 infection (Figure 5B). However, ZnO administration significantly down-regulated the mRNA level of *IL-1β* in the ileum and significantly up-regulated the mRNA level of *TNF-α* in the jejunum (Figure 5A,B).

### 3.6. ZnO Administration Dramatically Reduced the Intestinal Epithelial Cell Apoptosis in the Ileum of ETEC K88-Infected Piglets

**Figure 6:**
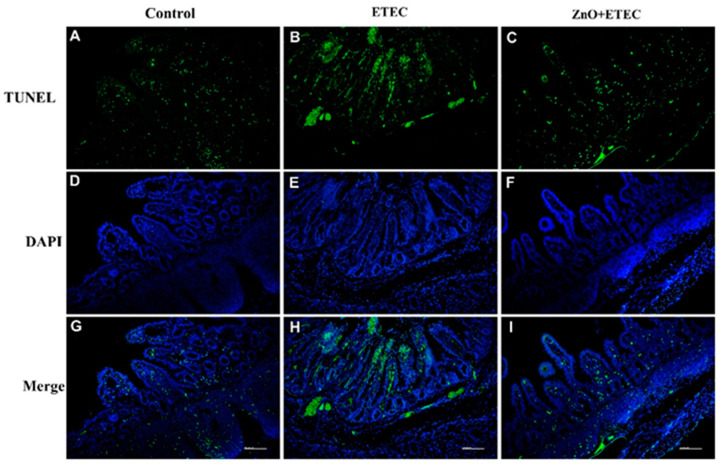
The effects of ZnO administration on the levels of apoptosis in the ileal epithelial cells of piglets infected with ETEC K88. (**A**,**D**,**G**) Control group; (**B**,**E**,**H**) ETEC group; (**C**,**F**,**I**) ZnO + ETEC group. TUNEL = terminal deoxynucleotidyl transferase dUTP nick end labeling; DAPI = 4,6-diamidino-2-phenylindole. Scale bar: 100 mm.

The effect of ZnO administration on the ileal epithelial cell apoptosis level in piglets infected with ETEC K88 is shown in Figure 6. Compared to the control group, the ileal epithelium apoptosis level was significantly increased in the ETEC K88-infected piglets (Figure 6A,B,D,E,G,H). However, compared with that in the ETEC group, ZnO administration significantly reduced apoptosis level in the ileal epithelium of ETEC K88-infected piglets (Figure 6C,F,I).

### 3.7. Effect of ZnO Administration on Intestinal Flora Structure in the ETEC K88-Infected Piglets

**Figure 7:**
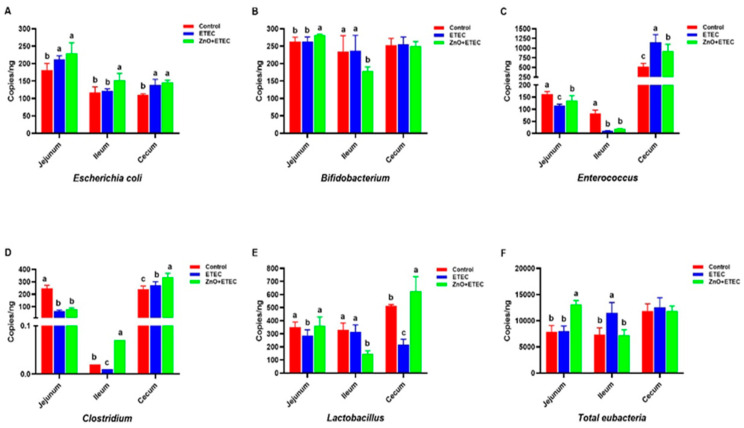
The effect of ZnO administration on the gut microbiota in piglets infected with ETEC K88. Data are shown as mean ± SD (n = 8), and mean values following different letters at the top of the a, b, and c plots signify statistically significant differences (*p* < 0.05).

Compared to the control group, the numbers of *Clostridium*, *Enterococcus*, and *Lactobacillus* in the jejunum (Figure 7C,D,E), *Enterococcus* and *Clostridium* in the ileum (Figure 7C,D), and *Lactobacillus* in the cecum (Figure 7E) were remarkably reduced after ETEC K88 infection. While the number of *Escherichia coli* in the jejunum and cecum (Figure 7A), *Enterococcus* and *Clostridium* in the cecum (Figure 7C,D), and the total number of bacteria in the ileum (Figure 7F) remarkably increased. However, the ZnO administration significantly increased the counts of *Lactobacillus*, *Bifidobacterium* and *Enterococcus* as well as the total number of bacteria in the jejunum (Figure 7B,C,E,F), *Escherichia coli* and *Clostridium* in the ileum (Figure 7A,D), *Clostridium* and *Lactobacillus* in cecum (Figure 7D,E) in ZnO + ETEC group. In addition, the count of *Bifidobacterium* in the ileum in the ZnO + ETEC group was significantly reduced (Figure 7B). In the jejunum, the total counts of *Bifidobacterium* and bacteria were not significantly affected after ETEC K88 infection (Figure 7B), which ZnO administration significantly increased the total counts of *Bifidobacterium* and bacteria in the ZnO + ETEC group (Figure 7B). ZnO administration reduced the increase in the count of total bacteria in piglet ileum infected with ETEC K88 (Figure 7F).

## 4. Discussion

Zinc (Zn) as an essential trace element plays an important role in maintaining the physiologic function, promoting metabolism, and ensuring healthy growth in animals [41]. The zinc required by animals can be supplemented in the form of ZnO. Previous studies have demonstrated that ZnO significantly influences the growth performance of animals and is closely related to the metabolism of certain nutrients. It can also improve the intestinal mucosal morphology of livestock and poultry, alleviate diarrhea, enhance immune function, and boost antioxidant enzyme activities [42,43]. Nonetheless, the effect of ZnO on the structure and function of the intestine and gut microbiota in piglets infected with ETEC K88 remains unexplored. The use of ZnO may effectively mitigate the symptoms of ETEC infection in piglets, potentially leading to a reduction in economic losses within the pig industry. This study showed that the ZnO administration alleviated diarrhea caused by ETEC K88 infection by improving intestinal morphology, restoring redox balance, reducing apoptosis of intestinal epithelial cells, and correcting gut microbiota dysbiosis in piglets.

ETEC K88 infection often results in watery diarrhea and reduced food intake, which can lead to intestinal damage in 7 to 15-day-old piglets [17,44]. Previous research has demonstrated that ETEC K88 decreased the natural barrier defenses of the intestinal mucosa and impaired intestinal mucosal function [45,46]. This study has shown that ZnO administration significantly improved the fecal scores of the ETEC K88-infected piglets (Figure 1), which provided new evidence for the positive efficacy of ZnO on the intestine of piglets infected with ETEC K88. Diamine oxidase (DAO) is stable in peripheral blood and is an intracellular enzyme in the small intestinal mucosa of mammals. Once the intestinal mucosa is damaged, the DAO in the small intestinal mucosa is released into the blood [47,48]. When the intestinal absorption function is impaired, the ability of the intestinal lumen to absorb D-xylose into the portal vein is impaired, which consequently lowers the level of D-xylose in the blood. Therefore, the intestinal mucosa integrity can be indirectly reflected by the activity of DAO and the level of D-xylose in the plasma, which is a key indicator for monitoring the intestinal barrier function [49,50]. Compared to the ETEC group, the activity of DAO in plasma in the ZnO + ETEC group was significantly reduced in this study (Figure 1). In addition, the intestinal epithelial cell renewal begins with a pool of intestinal mucosal crypt stem cells that form a new monolayer of epithelial cells, which then migrate to the intestinal villi and develop into mature cells capable of absorbing nutrients [51]. Therefore, the height of the intestinal mucosa villi is a key indicator of the gut health. The invasion of pathogenic microorganisms can accelerate epithelial cell regeneration, leading to the shortening of villi in the intestine [52]. The higher height of the villi means the more epithelial cells, the more surface area, and the better intestinal absorption of nutrients [53]. In this study, ZnO administration reduced the intestinal damage in ETEC K88-infected piglets by increasing the height of villi in the ileum, jejunum, and duodenum (Figure 3). In short, ZnO administration can alleviate the ETEC K88 infection-caused intestinal damage to relieve the piglet diarrhea.

Research has indicated that ETEC K88 may induce significant intestinal stress in piglets, including both oxidative and immune stress, which results in harm to the intestines [54,55]. The purpose of this study is to study the mechanism by which ZnO reduced intestinal damage in the ETEC K88-infected piglets, by measuring the redox levels of the intestine in the infected piglets. It is an important role for enzymatic and non-enzymatic antioxidant systems to maintain normal cellular activity in the organism, which can maintain a balance between pro-oxidant and antioxidant systems [56]. Intestinal oxidative stress induced by ETEC K88 infection is a key contributor to intestinal damage in piglets [57,58]. In the present study, ZnO administration enhanced the intestine antioxidant capacity of the ETEC K88-infected piglets (Figure 4). The T-SOD and CAT activities in the jejunum and T-SOD activity in the ileum were significantly increased after ZnO administration. Additionally, the level of MDA in the ileum and the content of H_2_O_2_ in the jejunum were significantly reduced, with a decreased trend observed in H_2_O_2_ content in the ileum (Figure 4). These results showed a new finding by which ZnO attenuated ETEC K88-induced intestinal damage in piglets. *IL-1β* is a key member of the IL-1 family, which has garnered attention for its important role in inflammation-related diseases59 [59]. *IL-1β* exhibits the activity of potent pro-inflammatory and promotes the secretion of various pro-inflammatory mediators, including chemokines and cytokines [60,61]. *IL-1β*, as a potent pro-inflammatory, is essential for the host defense response to infection, which is involved in the host response to a wide range of antigens and promotes the expression of inflammatory factors [62,63]. The mRNA level of *IL-1β* in the ileum was remarkably up-regulated after ETEC K88 infection (Figure 5B), which indicated that ETEC K88 infection stimulated the inflammatory response of the ileum in piglets in this study. However, the administration of ZnO remarkably suppressed the mRNA level of IL-1β in the ileum of the ETEC K88-infected piglets. In order to further study the underlying mechanisms, the apoptosis levels of intestinal epithelial cells were evaluated. The apoptosis levels of the ileal epithelial cells of piglets were increased after ETEC K88 infection; however, the addition of ZnO significantly reduced these apoptosis levels in piglets infected with ETEC K88 (Figure 6). Apoptosis is a form of programmed cell death, which plays a crucial role in maintaining the normal morphology and function of the intestine. The homeostatic imbalance between cell proliferation and apoptosis can adversely affect the normal structure and functionality of the intestine [64]. In summary, administering ZnO may help reduce the damage to the intestine in the ETEC K88-infected piglets. This is achieved by lowering the mRNA levels of *IL-1β* in the ileum and maintaining a balance between the apoptosis and regeneration of ileal epithelial cells.

The piglet intestine hosts a microflora that interacts symbiotically with the host. This microflora constitutes the microecological balance in the piglet intestine, which is essential for various physiological processes, including growth and development, disease resistance, and nutrient metabolism. Consequently, there has been an increasing focus on gut microflora research [65]. For instance, *Enterococcus* effectively inhibits intestinal pathogens and helps maintain intestinal health. Some studies have shown that *Enterococcus* can inhibit chlamydia and parasites, as well as prevent intestinal infections caused by protozoa [66,67,68]. *Clostridium* can alter the distribution of lymphocytes within the colorectal epithelium and promote the accumulation of Treg cells in the colonic mucosa, causing an increase in the levels of *TGF-β* in the colon [69,70]. *Lactobacillus* interacts with the immune system, selectively allowing *Lactobacillus* colonization, which helps regulate immune responses [71]. Additionally, *Lactobacillus* stabilizes the barrier function of the intestine by various mechanisms, including the promotion of mucus secretion and the upregulation of the levels of tight junction proteins [72]. The intestinal flora can be influenced by a whole range of factors, for example, genetics, host age, diseases, and environmental conditions [73,74,75]. The unstable gut microbial community is one of the primary causes of diarrhea for lactating piglets [76]. Therefore, it is vital to avoid the effect of external factors on the composition of intestinal flora to promote the health of the intestine in suckling piglets. Previous reports have indicated that low doses of encapsulated ZnO (500 mg Zn/kg) could modulate gut flora and improve the number of beneficial bacteria in the intestinal tract, reducing diarrhea in weaned piglets [77]. ETEC K88 notably increased the counts of *Enterococcus* in the cecum and the counts of total *eubacteria* in the ileum, while significantly decreasing the counts of *Enterococcus* and *Clostridium* in the ileum, the counts of *Lactobacillus* in the jejunum, and the counts of *Lactobacillus* in the cecum of piglets in this study. The above results suggested that ETEC K88 infection led to dysbiosis of intestinal flora in piglets. However, the addition of ZnO reversed the increases in the counts of *Enterococcus* in the cecum and the counts of total *eubacteria* in the ileum, as well as the decreases in the counts of *Clostridium* in the ileum, the counts of *Lactobacillus* and in the jejunum, and the counts of *Lactobacillus* in the cecum after ETEC K88 infection. In a word, incorporating ZnO into the feed alleviated the structural dysregulation of intestinal flora in the jejunum, ileum, and cecum of piglets infected with ETEC K88.

## 5. Conclusions

In conclusion, the administration of ZnO can mitigate diarrhea ETEC K88 infection- caused in piglets by reducing intestinal injury, decreasing epithelial cell apoptosis in the ileum, and modulating the gut microbiota. The present study offers a solid theoretical foundation for the application of ZnO in pig farming.

## Figures and Tables

**Table 1:** Primers sequences.

Strains	Forward (5′–3′)	Reverse (5′–3′)
*Bifidobacterium*	TCGCGTC(C/T)GGTGTGAAAG	CCACATCCAGC(A/G)TCCAC
*Enterococcus*	CCCTTATTGTTAGTTGCCATCATT	ACTCGTTGTACTTCCCATTGT
*Lactobacillus*	AGCAGTAGGGAATCTTCCA	CACCGCTACACATGGAG
*Escherichia coli*	CATGCCGCGTGTATGAAGAA	CGGGTAACGTCAATGAGCAAA
*Clostridium*	AATGACGGTACCTGACTAA	CTTTGAGTTTCATTCTTGCGAA
Total eubacteria (16S rRNA)	CGGTCCAGACTCCTACGGG	TTACCGCGGCTGCTGGCAC

**Table 2:** Sequences of the primers.

Gene	Forward (5′–3′)	Reverse (5′–3′)
*TNF-α*	TCCAATGGCAGAGTGGGTATG	AGCTGGTTGTCTTTCAGCTTCAC
*IL-1β*	CAACGTGCAGTCTATGGAGT	GAGGTGCTGATGTACCAGTTG
*RPL4*	GAGAAACCGTCGCCGAAT	GCCCACCAGGAGCAAGTT
*IL-6*	TACTGGCAGAAAACAACCTG	GTACTAATCTGCACAGCCTC

## Data Availability

The raw data supporting the conclusions of this article will be made available by the authors, without undue reservation.

## Supplementary Materials

The following supporting information can be downloaded at: https://www.mdpi.com/article/10.3390/vetsci12020115/s1, Figure S1 The effects of ZnO administration on ADFI and Body weight in ETEC K88-infected piglets. (A) Effect of ZnO administration on ADFI of ETEC K88-infected piglets. (B) Effect of ZnO administration on Body weight in ETEC K88-infected piglets. Data are presented as mean ± SD (n = 8), and mean values following different letters at the top of the ^a, b, c^ plots indicated statistically significant differences (*p* < 0.05).

## Abbreviations

ADFI	Average Daily Feed Intake
AST	Aspartate aminotransferase
BASO	Basophil
BUN	Blood Urea Nitrogen
CAT	Catalase
CHCM	Cell Hemoglobin Concentration Mean
CK	Creatine Kinase
CREA	Creatinine
DAO	Diamine oxidase
ETEC	Enterotoxigenic Escherichia coli
GGT	Gamma-Glutamyl Transferase
GSH-Px	Glutathione peroxidase
H_2_O_2_	Hydrogen peroxide
HDL	High-Density Lipoprotein
HDW	Hemoglobin Distribution Width
*IL-1β*	Interleukin-1β
*IL-6*	Interleukin-6
LDL	Low-Density Lipoprotein
LUC	Large Unstained Cells
LYMPH	Lymphocytes
MCHC	Mean Corpuscular Hemoglobin Concentration
MDA	Malondialdehyde
MONO	Monocytes
MPO	Myeloperoxidase
NEUT	Neutrophils
*TNF-α*	Tumor Necrosis Factor Alpha
T-SOD	Total Superoxide Dismutase
WBC	White Blood Cell
Zn	Zinc
ZnO	Zinc oxide
