# Supplementary material for: Zinc Oxide Administration Relieves the Diarrhea of ETEC K88-Infected Piglets by Reducing Ileal Apoptosis and Maintaining Gut Microbial Balance
Source: Vet Sci. 2025 Feb 2;12(2):115. doi: 10.3390/vetsci12020115 (PMC11861302; doi:10.3390/vetsci12020115)
Supplement: Supplementary file 1 [file vetsci-12-00115-s001.zip › vetsci-3403033-supplementary.pdf]

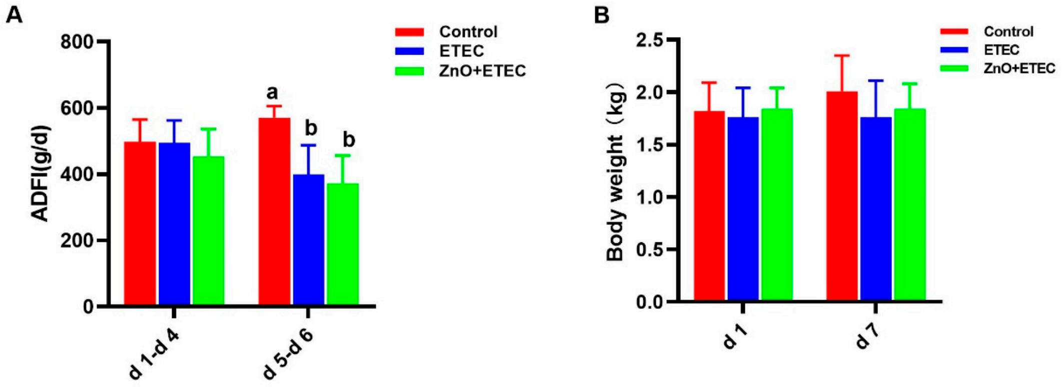

Figure S1. The effects of ZnO administration on ADFI and Body weight in ETEC K88-infected piglets. (A) Effect of ZnO administration on ADFI of ETEC K88-infected piglets. **(B)** Effect of ZnO administration on Body weight in ETEC K88-infected piglets. Data are presented as mean ± SD (n = 8), and mean values following different letters at the top of the $^{a, b, c}$ plots indicated statistically significant differences ($p < 0.05$).
